# Inter-rater reliability in performing stifle goniometry in normal and cranial cruciate ligament disease affected dogs: a prospective randomized controlled study

**DOI:** 10.1186/s12917-024-04206-5

**Published:** 2024-08-01

**Authors:** Frederik Volz, Johannes Maximilian Schmutterer, Tanja Stephanie Vockrodt, Yury Zablotski, Susanne Katja Lauer

**Affiliations:** 1 LMU Small Animal Clinic, Centre for Clinical Veterinary Medicine, Ludwig-Maximilians-Universität München, Veterinärstraße 13, 80539 Munich, Germany; 2 Tierklinik Dinkelsbühl, Heininger-Ring 17, 91550 Dinkelsbühl, Germany

**Keywords:** Cranial cruciate ligament disease, Compliance, Dog, Goniometry, Reliability

## Abstract

**Background:**

Goniometry can be performed clinically in dogs with cranial cruciate ligament disease (CCLD). The purpose of this study was (1) to compare reliability of stifle goniometry in dogs with CCLD and healthy dogs and (2) to investigate the effect of compliance on measurements. Dogs presented for surgical intervention for CCLD (CCL-Dogs; *n* = 15) and orthopedically healthy dogs (C-Dogs; *n* = 11) were enrolled in this prospective randomized controlled trial. In each dog, three observers randomly measured maximum stifle flexion (mSF) and maximum stifle extension (mSE) three times with a standard goniometer with the scale covered, while dog compliance was scored (Scores: C0: excellent - C4: poor). Intraclass correlation coefficient (ICC) was calculated for intra-/interobserver reliability. Effects on measurements were evaluated with mixed-effect models (MEM).

**Results:**

Maximum stifle extension and mSE-compliance were significantly decreased in CCL-Dogs compared to C-Dogs (*p* ≤ 0.004), but mSF and mSF-compliance did not differ between groups. Intraobserver reliability was excellent for all dogs during mSE (ICC:0.75–0.99) and mSF (ICC:0.89–0.99). Interobserver reliability was excellent for mSF in both groups (ICC: C-Dogs:0.84, CCL-Dogs:0.9) and for mSE in CCL-Dogs (ICC:0.94) but only fair for mSE in C-Dogs (ICC:0.58). Robust MEM showed that the combined average of all mSE measurements of all three observers was affected by compliance in both groups (*p* < 0.001). This effect was not observed for single mSE-measurements by themselves.

**Conclusion:**

The results of this study indicate that compliance may affect goniometric stifle extension measurements in healthy and CCLD dogs. In a clinical setting, intra-/interobserver reliability was excellent for all measurements except for maximum stifle extension in healthy dogs.

**Supplementary Information:**

The online version contains supplementary material available at 10.1186/s12917-024-04206-5.

## Background

In human medicine, goniometry has been employed to measure joint angles since the 1970s and has established itself as a fundamental tool in physical therapy and orthopedics [1, 2]. In veterinary medicine, joint range of motion (ROM) can be either measured actively using kinematic gait analysis [3] or passively via goniometric measurements [4, 5]. Kinematic gait analysis is still primarily utilized in veterinary research laboratories and rarely employed for objective gait analysis in the clinical setting due to high cost, limited user friendliness and high expenditure of time [3, 6, 7]. In contrast to kinematic gait analysis, standard goniometry has become a well-established field test in veterinary medicine [4, 8]. Standard goniometric measurements of joints are commonly performed to assess joint range of motion (ROM) and to ascertain any restrictions in mobility or joint function of dogs in canine physiotherapy [9, 10], but are also employed as outcome parameter in canine orthopedic research [9, 11–17]. Although primarily performed in dogs, goniometry is also reported in cats, calves, and horses [18–20]. Goniometric measurements based on anatomic landmarks with standard plastic goniometer are easy to learn, time-efficient and inexpensive. [5, 9, 21]. Variability of standard goniometric measurements was not documented to be improved using smartphone-based or digital goniometers compared to plastic goniometers [21, 22]. Low intra-/intertester variability was determined for goniometric measurements of large joints in healthy Labrador Retrievers and measurements did not differ between awake or sedated dogs [5]. Similarly, variability was low for goniometric measurements of carpal, tarsal, elbow, stifle, shoulder and hip joint range of motion (ROM) in healthy sedated German Shepherd Dogs [21]. However, the conformation of dogs, which varies between breeds, can have a significant influence on goniometric measurements [23].

Standard goniometric measurements have been routinely employed in numerous orthopedic studies on dogs suffering from elbow or hip dysplasia, shoulder instability, proximal humeral osteochondrosis or CCLD to evaluate outcome [9, 10, 12–17, 24]. Multiple studies have shown that stifle extension is decreased in dogs with CCLD [25–27]. A retrospective study on sedated dogs with CCLD showed that the reduction of goniometric stifle ROM was primarily due to reduced stifle extension angles, while flexion was frequently not affected [27]. Interestingly, in 13.7% of these dogs goniometric stifle extension and flexion were normal. The authors stated, that goniometric measurements were performed under sedation to eliminate pain associated with capsular stretching and increased nociceptor activity during maximum joint range of motion, as well as restrictions due to muscular contractures [27]. Stifle extension and flexion are frequently associated with discomfort during orthopedic examination of dogs with CCLD [28]. Patient compliance affected by potential discomfort during joint manipulation is currently not routinely quantitatively documented in small animal practice and a validated scoring system has not been established yet [29]. The goniometric measurements in awake dogs may be impaired due to insufficient patient compliance when stifles are manipulated by the examiner. This is supported by one feline study showing differing goniometric ROM of osteoarthritic stifles when measured in awake versus sedated cats [30]. The influence of sedation on goniometric measurements in dogs remains unclear as there are conflicting results in the literature. While in healthy Labrador Retrievers no differences among 15 measurements between sedated or awake dogs are reported [5], there are also reports of significant alterations for goniometric measurements of the elbow joint in dogs with elbow osteoarthritis under general anesthesia or sedation [31]. Multiple studies performed goniometric measurements while dogs were sedated [12, 14, 15, 21, 23, 27]. In these and other clinical studies, typically one to three replicant measurements were performed in extension and flexion of the affected joints by one to three observers in either sedated or awake dogs [5, 9, 12–17, 21–24, 27, 32].

To the authors’ knowledge, reliability of standard goniometric measurements to evaluate ROM has not been established yet for diseased stifle joints with CCLD. We hypothesized (1) that the reliability of stifle goniometric measurements would not differ between dogs with CCLD and orthopedically healthy dogs and (2) that goniometric stifle measurements would be affected by dog compliance.

## Results

The CCL-Dogs consisted of five mixed breed dogs, five Labrador Retrievers and one of each of the following breeds: White Swiss Shepherd, Breton, Old English Bulldog, Doberman and Beagle. Seven dogs were males (three neutered and four intact) and eight were females (seven spayed and one intact). One dog in the CCL-Dogs had a compliance score of four and only one out of the three observers performed all measurements. In this dog, observer two performed all three flexion and only one stifle extension measurement, while observer 3 performed no measurements. None of the other dogs showed any compliance scores > 2 during goniometric measurements. None of the dogs showed any vocalizing or flinching during the measurements. Compliance scores per dog, per observer and performed measurement are displayed in Appendix 1.

The C-Dogs consisted of three Labrador Retrievers, three German Shepherds and one of each of the following breeds: Mixed breed, Australian Shepherd, Rhodesian Ridgeback, Malinois and German Shorthair. Seven dogs were males (three neutered and four intact) and four were females (two spayed and two intact).

Mean body weight was 28.7 ± 4.9 kg for the C-Dogs and 30.4 ± 6.6 kg for the CCL-Dogs. Median BCS was 4/9 for the C-Dogs (range: 4/9 − 6/9) and 5/9 (range: 4/9 − 8/9 for the CCL-Dogs. Mean age was 3.8 ± 1.7 years for the C-Dogs and 7.2 ± 2,9 years for the CCL-Dogs.

There was no significant difference for sex, body weight and BCS between C-Dogs and CCL-Dogs. The dogs in the control group were significantly younger than CCL-Dogs (*p* = 0.004). Maximum stifle flexion angles and dog compliance during stifle flexion measurements did not differ significantly between groups (Tables 1 and 2). Stifle extension was significantly decreased in CCL-Dogs compared to C-Dogs (Table 1). Compliance scores during stifle extension were significantly higher in CCL-Dogs compared to C-Dogs indicating less compliance in CCL-Dogs (Table 2). This result was observed for each single observer and all observers as a group (Tables 1 and 2).

**Table 1:** Mean ± SD goniometric maximum stifle flexion and extension measurements in control dogs (C-Dogs) and in dogs with cranial cruciate ligament disease (CCL-Dogs)

	Maximum stifle flexion (°)			Maximum stifle extension (°)		
	C-Dogs	CCL-Dogs	p - value	C-Dogs	CCL-Dogs	p - value
Observer 1 (Intern)	39.1 ± 4.7	47.5 ± 9.8	0.26	152 ± 5.4	139 ± 12	< 0.001
Observer 2 (Diplomate)	41.1 ± 4.0	44.4 ± 7.5	0.22	146 ± 4.8	141 ± 6.8	0.04
Observer 3 (Resident)	36.5 ± 3.8	43.7 ± 10.7	0.2	159 ± 4.5	149 ± 8.9	0.003
All observers	38.9 ± 3.4	44.2 ± 8.7	0.13	152 ± 3.4	140 ± 17	< 0.001

**Table 2:** Comparison of median (range) compliance scores during goniometric measurements for each observer in control dogs (C-dogs) and dogs with cranial cruciate ligament disease (CCL-Dogs)

	Compliance scores (maximum stifle flexion)			Compliance scores (maximum stifle extension)		
	C-Dogs	CCL-Dogs	p - value	C-Dogs	CCL-Dogs	p - value
Observer 1 (Intern)	0 (0–1)	0 (0–2)	0.33	0 (0–1)	1 (0–3)	0.027
Observer 2 (Diplomate)	0 (0)	0 (0–1)	0.26	0 (0–1)	1 (0–4)	0.041
Observer 3 (Resident)	0 (0)	0 (0–4)	0.41	0 (0)	1 (0–4)	0.047
All observers	0 (0–1)	0 (0–4)	0.16	0 (0–1)	1 (0–4)	0.004

For C-Dogs, interobserver reliability was excellent for maximum stifle flexion (ICC = 0.84) and fair for maximum stifle extension (ICC = 0.58). For CCL-Dogs, interobserver reliability was excellent for maximum stifle flexion (ICC = 0.9) and stifle extension (ICC = 0.94). Intraobserver reliability for each observer was excellent in both groups (Table 3).

**Table 3:** Intraobserver reliability: Intraclass correlation coefficients of each observer for goniometric flexion and extension measurements in control dogs (C-Dogs) and dogs with cranial cruciate ligament disease (CCL-Dogs)

Observer	ICC Flexion C-Dogs	ICC Flexion CCL-Dogs	ICC Extension C-Dogs	ICC Extension CCL-Dogs
Observer 1 (Intern)	0.87	0.93	0.76	0.92
Observer 2 (Diplomate)	0.91	0.96	0.75	0.81
Observer 3 (Resident)	0.9	0.99	0.81	0.99

ICC: Intraclass correlation coefficient

In the mixed-effect-models (MEM) the first stifle flexion measurement was significantly affected by the observer with an estimate effect ranging from 3.18° to 4.34° (*p* = 0.004–0.029), but not by BCS, group or compliance score. The second stifle flexion measurement was significantly affected by the observer with an estimate effect between 1.69° − 3.18° (*p* = 0.023), but not by BCS, group or compliance score. No significant effect was observed for the third flexion measurement for any of the factors based on MEM.

The first stifle extension measurement was significantly affected by group with an estimate effect of 10.62° (*p* < 0.001) and by the observers with an estimate effect between 1.13° – 9.86° (*p* < 0.001). The second stifle extension measurement was also significantly affected by group with an estimate effect of 10.25° (*p* = 0.003) and by the observers with an estimate effect between 3.12° – 8.09° (*p* < 0.001). The third stifle extension measurement was significantly affected by group with an estimate effect of 9.98° (*p* < 0.001) and of the observers with an estimate effect 0.41–6.43°(*p* = 0.002). Body conditioning score and dog compliance score did not significantly affect any single measurement of stifle extension.

Based on the robust MEM, stifle extension measurements were significantly affected by dog compliance with an estimate effect of 10.71° (*p* < 0.001). However, dog compliance did not affect stifle flexion measurements (*p* = 0.722) in the robust MEM.

## Discussion

Despite its utilization as an outcome measurement tool in clinical studies, reliability of goniometric measurements has not been assessed in dogs suffering from CCLD. In the present study, the reliability of stifle goniometry was evaluated in awake dogs with CCLD. We hypothesized that the reliability of stifle goniometric measurements would not differ between dogs with CCLD and orthopedically healthy dogs. This hypothesis was partially confirmed, as intraclass correlation coefficients indicated an excellent intra- and interobserver reliability for almost all goniometric stifle measurements in both groups, except for merely fair interobserver reliability for extension measurements in healthy dogs.

Further, we hypothesized that goniometric stifle measurements would be affected by dog compliance. This hypothesis was partially confirmed, as dog compliance affected stifle extension significantly in all dogs, but did not affect stifle flexion measurements. Moreover, this could only be demonstrated in the robust MEM, but not in the MEMs of the single measurements.

The merely fair interobserver reliability during stifle extension measurements in healthy dogs, observed in our study, contrasts with a previous goniometric study on healthy Labrador Retrievers [5]. This discrepancy may be explainable by the lower number of replicant goniometric measurements performed in our study, probably resulting in less data variability. Our study was conducted to reflect upon the typical use of goniometry in dogs with CCLD under clinical field conditions. Thus, we only performed three replicant measurements per joint position, as suggested in the literature [4]. However, in the aforementioned study [5], each observer performed five sets of three replicant measurements per dog and joint position. Moreover, in contrast to our study, goniometric measurements performed in awake and sedated dogs were combined, as no significant differences could be detected between measurements in sedated and awake dogs. As half of the measurements were performed in sedated dogs, measurements were most probably more consistent due to missing muscle contraction/density under sedation and dog temperament affected measurements potentially to a lesser degree [5].

The lower number of healthy dogs evaluated in our study may have also affected variability and interobserver reliability negatively. From a purely subjective point of view, the observers gained the impression that healthy dogs appeared to experience a more pronounced acclimatization throughout the measurements. Healthy dogs frequently appeared tenser compared to the CCL-Dogs throughout the first measurements and then became progressively more relaxed throughout the remainder of the measurements. This finding may be explainable by the fact that the C-Dogs were significantly younger compared to the CCL-Dogs and had potentially undergone less training and may have been less composed during the measurements compared to the older CCL-Dogs.

Furthermore, the slightly differing stifle extension and flexion observed in our study is most probably normal for our heterogenous study population including multiple dog breeds with differing conformation. Our results are consistent with a previous study by Sabanci and Ocal [23]documenting stifle ROM in healthy dogs, but differ slightly from standard values established for healthy Labrador Retrievers [5].

Similar to our study on awake dogs, a previous goniometric study on sedated dogs with CCLD also showed that stifle ROM was primarily reduced due to loss of stifle extension [27]. As loss of goniometric stifle extension was also observed in sedated dogs with CCLD [27], discomfort during stifle extension may not be a crucial factor compromising reliability of goniometric measurements in dogs with CCLD. However, this is not supported by a recent study showing that in dogs with elbow osteoarthritis, sedation or general anaesthesia significantly increased goniometric measured elbow flexion and extension angles [31]. A study design allowing for the direct comparison of goniometric measurements of stifle ROM performed in awake and under sedation in dogs with CCLD is needed to further investigate this observation.

The decreased dog compliance observed in CCL-dogs compared to healthy dogs during goniometric measurement of stifle extension is indicative for discomfort throughout this manoeuvre. A similar response is also observed, when stifle extension is performed during the orthopedic examination of dogs with CCLD [28]. Particularly in dogs with partial cranial cruciate ligament tears, a painful reaction is commonly only noted upon stifle extension [28]. Although dog compliance did not affect single goniometric measurements in our study, the robust MEM on all measurements as a whole demonstrated a significant effect of dog compliance on stifle extension. Thus, dog compliance should be taken into consideration as a factor affecting goniometric stifle extension measurements in awake dogs with discomfort due to CCLD. In studies utilizing goniometric stifle extension as outcome measure in dogs with stifle disease, sedation during goniometry may be beneficial to circumvent the effect of potentially varying dog compliance at different time points. On the other hand, clinicians need to carefully weigh risks and benefits of sedation for each individual patient undergoing serial goniometric measurements.

The effect of dog disposition on compliance was not evaluated in our study and the impact of sedation or general anaesthesia on goniometric measurements in dogs with CCL disease has not been investigated yet. Further prospective comparative studies are needed to evaluate the effect of pain medications, sedation and anaesthesia on compliance of dogs with differing stifle disorders undergoing standard goniometric evaluation.

Based on the MEMs of the single measurements, some of the goniometric extension and flexion measurements were affected by the observer. Nevertheless, based on the ICC, reliability was excellent and the estimate effect of the observer in our results was close to the previously reported variability between observers with a range of 1–6° [5]. This slightly higher variability is explainable, as we investigated a clinical scenario comprising a lower number of goniometric measurements per observer, that still deems acceptable in clinical patients [4].

This study has several limitations, the small sample size was attributable to the fact that one observer left the clinic during the study period and a new observer deemed not acceptable for statistical reliability evaluation. This resulted in underpowering of the study according to the a priori power analysis. Furthermore, non-parametric tests had to be used for statistical analysis, as the data were not normally distributed in our study. Consequently to these circumstances, we performed robust MEM`s with the patient number as random effect to minimize the influence of some outliners in the data. Another relative limitation is the heterogeneity of the dogs included in our study. As all dogs were clinical patients, breed, sex, purpose, body condition, muscularity and disposition differed in the dogs included in our study. This may have impacted our data, as breed, body weight and hind limb muscularity have been shown to affect the goniometric stifle flexion, extension angle and ROM in healthy dogs [23]. Additionally, osteoarthritic changes on tibia and femur may have affected the identification of goniometric landmarks during the measurements. Furthermore, the standardized order of the flexion and then extension measurements may have influenced the results, as stifle extension is more often painful during clinical examinations [28]. Finally, stifle radiographs could not be performed in the healthy control dogs due to national animal welfare regulations. Thus, we cannot exclude completely that dogs included in the healthy control group, may have had minor degenerative stifle joint disease or other subtle orthopedic diseases, that could not be identified based on history, orthopedic examination, and objective gait analysis.

## Conclusion

In conclusion, in a clinical setting with three replicant goniometric measurements, intra- and interobserver reliability for stifle goniometry in Dogs with CCLD was excellent. However, in this clinical setup, interobserver reliability for stifle extension measurements in healthy dogs was only fair. Dog compliance may impact goniometric stifle extension measurements in non-sedated dogs with CCLD and in healthy dogs. Thus, dog compliance should be taken into consideration in studies utilizing serial stifle goniometric measurements to evaluate outcome.

## Methods

### Study design and ethics

This prospective randomized controlled study was conducted with a simple randomization at a veterinary teaching hospital. The study protocol was approved by the Ethical Committee of the Faculty of Veterinary Medicine, LMU Munich (approval number: 177-05-06-2019). An informed client consent was obtained from all owners for the participation of their dogs in this study prior to enrollment.

### Dogs

Privately owned dogs with CCLD presented for tibial plateau leveling osteotomy (TPLO) and orthopedically healthy dogs presented for health checks at the LMU Small Animal Clinic at Ludwig Maximilians University in Munich were recruited for this study. Inclusion criteria were a body weight between 20 and 40 kg and a body conditioning score (BCS) between 4/9 and 6/9 [33]. The dogs with CCLD (CCL-dogs) could only be included, if they had no other orthopedic or neurological disorders based on history, general exam, orthopedic exam and objective kinetic gait analysis. Only dogs without evidence of musculoskeletal abnormalities upon history, physical examination, orthopedic examination and objective kinetic gait analysis were included in this study for the control group (C-Dogs). Dogs were excluded if they did not tolerate the goniometric measurement at all or if the compliance score was four, according to the modified compliance scale (Table 4).

Orthogonal radiographs of the affected stifle were performed in all CCL-dogs. Standard goniometric measurements were performed in awake CCL-dogs prior to stifle magnetic resonance imaging (MAGNETOM Symphony 1.5 Tesla, Fa. Siemens Healthcare GmbH, Erlangen, Germany) and TPLO. For the diagnosis of CCLD, the following magnetic resonance imaging sequences were obtained: T1-weighted in the sagittal plane, proton density weighted with fat saturation in dorsal and sagittal planes, and T2-weighted in the sagittal plane. In total, 26 client-owned dogs participated in the study. Fifteen dogs with CCLD and 11 orthopedically healthy dogs were included.

### Goniometric measurements

Three observers with different experience levels (observer 1: surgical intern, observer 2: board-certified surgeon and Diplomate in Veterinary Sports Medicine and Rehabilitation, observer 3: third year surgical resident) performed standard goniometric measurements. Prior to the study, all three observers discussed how to determine the goniometric landmarks according to a previous study [5]. Landmark determination was then trained by all observers together on three clinical patients (healthy and with CCLD).

Goniometric measurements were performed by the observers in a randomized order based on a randomizer (Microsoft Excel, Version 16.65, Microsoft Coroperation, Redmond, WA, USA). The scale of a standard plastic goniometer (Goniometer, KaWe - KIRCHNER & WILHELM GmbH + Co. KG., Asperg, Germany) was covered with black obscure tape to blind the observers to the goniometric measurements. After an acclimatization time in the exam room, the dogs were placed in lateral recumbency. In CCL-dogs, the affected stifle was placed in upper position. In C-dogs, one stifle was randomly assigned and placed in upper position. During the measurements, each dog was immobilized by an assistant for all three observers. Goniometric measurements of the stifle extension and flexion were performed as previously described [5]. Stifle joint flexion and extension were measured as the angle between the tibial shaft and the longitudinal axis of the femur, defined as the line that joined the lateral femoral epicondyle and the greater trochanter [5]. Additional chalk marks could be placed on the skin, for flexion and extension measurements separately, at discretion of each observer. Each observer performed three measurements of stifle flexion initially, followed by measurements of stifle extension in maximal comfortable flexion (mSF) or extension (mSE), respectively. Each observer performed all goniometric flexion measurements first and then the extension measurements. Compliance scores for the dogs were documented by the observer for each single measurement according to a pain scale for joint manipulations in cats [34]. The compliance scale was modified and ranged from 0 to 4 (Table 4).

**Table 4:** Compliance score scale modified from Benito, Gruen [34] for goniometric stifle flexion and extension measurements

Compliance scores	
Compliance score 0	No response during measurement
Compliance score 1	Mild response, mild body tension, licking, smacking lips
Compliance score 2	Moderate response, more body tension, orient to site (dog is orientating to the examiner or the measured joint), may vocalise
Compliance score 3	Orients to site, forcible withdrawal, flinching, vocalizing
Compliance score 4	Dog did not tolerate measurements

After each single measurement, the goniometer was placed on white paper and the angle from the goniometer was copied on separate sheets for each measurement by the observer. Any chalk marks were removed from the skin between observers and dogs were allowed to rest and walk for at least 10 min prior to the measurements of the next observer. The mSE and mSF measurements copied on the paper were later measured by one single author with a digital angle gauge (with 0.1° increments) to record the angle degree for each measurement.

### Statistical analysis

Data were analyzed with commercial statistical software (SPSS, IBM SPSS Statistics Version 27 and R Version 4.3.3 (2024-02-29). The continuous data were evaluated using the Shapiro-Wilk test for normal distribution. If they were not normally distributed, non-parametric tests were used. Intraclass correlation coefficients (ICC) were calculated for the goniometric measurements to determine intra-/interobserver reliability. An ICC < 0.4 was regarded as poor, from 0.4 to 0.59 as fair, from 0.6 to 0.75 as good and > 0.75 as excellent reliability as previously described [35].

Initially, six linear mixed-effects models were constructed using restricted maximum likelihood (REML) estimation and the nloptwrap optimizer package for statistical software (R): three models aimed at predicting stifle flexion angles and three for stifle extension angles. Each model included four predictors: Observer, group association CCL-dog/C-dog, BCS, and compliance score during each measurement. Model assumptions [36] were verified for all models, encompassing: (1) normality of residuals assessed by the Shapiro-Wilk test and visual examination of residual distributions, (2) evaluation of multicollinearity among predictors using the Variation Inflation Factor (VIF), (3) assessment of heteroscedasticity (constancy of error variance) via the Breusch and Pagan test [37], and (4) identification of outliers and influential points using Cook’s Distance [38].

Additionally, two further linear mixed-effects models were performed to estimate the mean stifle extension and flexion angles across all observers, incorporating the mean Compliance Scores. Robust mixed-effects models were selected due to the presence of outliers and influential points [39]. Patient number was included as a random effect in all models. A significance level of *p* ≤ 0.05 was applied to all analyses.

A priori power analysis was performed with values from previous studies using G*Power [40]. Final analysis was performed with; effect size d = 0.65, α = 0.05 and power = 0.8 and revealed a total sample size of 60 dogs.

## Electronic supplementary material

Below is the link to the electronic supplementary material.

Supplementary Material 1

## Data Availability

The datasets used and analysed during the current study are available from the corresponding author upon reasonable request.

## Abbreviations

BCS: Body condition Score
CCL-Dogs: Group of dogs with cranial cruciate ligament disease
CCLD: Cranial Cruciate Ligament disease
C-Dogs: Control dogs group
ICC: Intraclass correlation coefficient
MEM: Mixed effect models
mSE: Maximum comfortable stifle extension
mSF: Maximum comfortable stifle flexion
REML: Restricted maximum likelihood
ROM: Range of motion
VIF: Variation Inflation Factor
