# Supplementary material for: Inter-rater reliability in performing stifle goniometry in normal and cranial cruciate ligament disease affected dogs: a prospective randomized controlled study
Source: BMC Vet Res. 2024 Aug 1;20:339. doi: 10.1186/s12917-024-04206-5 (PMC11293097; doi:10.1186/s12917-024-04206-5)
Supplement: Supplementary file 1 — Supplementary Material 1 [file 12917_2024_4206_MOESM1_ESM.docx]

| Dog Number | Group | Observer 1 (Intern) | | | | | |
| --- | --- | --- | --- | --- | --- | --- | --- |
|  |  | Compliance Score Flexion 1 | Compliance Score Flexion 2 | Compliance Score Flexion 3 | Compliance Score Extension 1 | Compliance Score Extension 2 | Compliance Score Extension 3 |
| 1 | CCL-Dogs | 0 | 0 | 0 | 0 | 0 | 0 |
| 2 | CCL-Dogs | 1 | 1 | 1 | 1 | 1 | 1 |
| 3 | CCL-Dogs | 0 | 0 | 1 | 2 | 2 | 2 |
| 4 | CCL-Dogs | 3 | 1 | 2 | 3 | 2 | 2 |
| 5 | CCL-Dogs | 0 | 0 | 0 | 1 | 1 | 1 |
| 6 | CCL-Dogs | 0 | 0 | 0 | 1 | 1 | 1 |
| 7 | CCL-Dogs | 0 | 1 | 0 | 0 | 0 | 0 |
| 8 | CCL-Dogs | 0 | 0 | 0 | 0 | 0 | 0 |
| 9 | CCL-Dogs | 0 | 0 | 0 | 0 | 0 | 0 |
| 10 | CCL-Dogs | 0 | 0 | 0 | 1 | 0 | 0 |
| 11 | CCL-Dogs | 0 | 0 | 0 | 2 | 1 | 2 |
| 12 | CCL-Dogs | 1 | 2 | 0 | 1 | 0 | 0 |
| 13 | CCL-Dogs | 0 | 0 | 1 | 0 | 0 | 1 |
| 14 | CCL-Dogs | 0 | 0 | 0 | 2 | 2 | 1 |
| 15 | CCL-Dogs | 0 | 0 | 0 | 2 | 1 | 1 |
| 16 | C-Dogs | 0 | 0 | 0 | 0 | 0 | 0 |
| 17 | C-Dogs | 0 | 0 | 0 | 0 | 0 | 1 |
| 18 | C-Dogs | 0 | 0 | 0 | 1 | 1 | 1 |
| 19 | C-Dogs | 0 | 0 | 0 | 0 | 0 | 0 |
| 20 | C-Dogs | 0 | 0 | 0 | 0 | 0 | 0 |
| 21 | C-Dogs | 0 | 0 | 0 | 1 | 1 | 0 |
| 22 | C-Dogs | 0 | 0 | 0 | 0 | 0 | 0 |
| 23 | C-Dogs | 0 | 0 | 0 | 0 | 0 | 0 |
| 24 | C-Dogs | 0 | 1 | 1 | 0 | 0 | 1 |
| 25 | C-Dogs | 0 | 0 | 0 | 0 | 0 | 0 |
| 26 | C-Dogs | 1 | 0 | 0 | 0 | 0 | 0 |

Appendix 1 Compliance Sores per Observer per Measurement per Dog

| Dog Number | Group | Observer 2 (Diplomate) | | | | | |
| --- | --- | --- | --- | --- | --- | --- | --- |
|  |  | Compliance Score Flexion 1 | Compliance Score Flexion 2 | Compliance Score Flexion 3 | Compliance Score Extension 1 | Compliance Score Extension 2 | Compliance Score Extension 3 |
| 1 | CCL-Dogs | 0 | 0 | 0 | 1 | 1 | 1 |
| 2 | CCL-Dogs | 2 | 2 | 0 | 0 | 1 | 1 |
| 3 | CCL-Dogs | 1 | 1 | 1 | 2 | 2 | 1 |
| 4 | CCL-Dogs | 1 | 1 | 2 | 3 | 4 | 4 |
| 5 | CCL-Dogs | 0 | 0 | 0 | 1 | 0 | 0 |
| 6 | CCL-Dogs | 0 | 0 | 0 | 1 | 1 | 0 |
| 7 | CCL-Dogs | 0 | 0 | 0 | 0 | 0 | 0 |
| 8 | CCL-Dogs | 0 | 0 | 0 | 1 | 0 | 0 |
| 9 | CCL-Dogs | 0 | 0 | 0 | 0 | 0 | 0 |
| 10 | CCL-Dogs | 0 | 0 | 0 | 0 | 0 | 0 |
| 11 | CCL-Dogs | 0 | 0 | 0 | 0 | 0 | 0 |
| 12 | CCL-Dogs | 0 | 0 | 0 | 0 | 0 | 0 |
| 13 | CCL-Dogs | 0 | 0 | 0 | 0 | 0 | 0 |
| 14 | CCL-Dogs | 0 | 1 | 1 | 0 | 0 | 0 |
| 15 | CCL-Dogs | 0 | 0 | 0 | 0 | 1 | 0 |
| 16 | C-Dogs | 0 | 0 | 0 | 0 | 0 | 0 |
| 17 | C-Dogs | 0 | 0 | 0 | 0 | 0 | 0 |
| 18 | C-Dogs | 0 | 0 | 0 | 1 | 0 | 0 |
| 19 | C-Dogs | 0 | 0 | 0 | 0 | 0 | 0 |
| 20 | C-Dogs | 0 | 0 | 0 | 0 | 0 | 0 |
| 21 | C-Dogs | 0 | 0 | 0 | 0 | 0 | 0 |
| 22 | C-Dogs | 0 | 0 | 0 | 0 | 0 | 0 |
| 23 | C-Dogs | 0 | 0 | 0 | 0 | 0 | 0 |
| 24 | C-Dogs | 0 | 0 | 0 | 0 | 0 | 0 |
| 25 | C-Dogs | 0 | 0 | 0 | 0 | 0 | 0 |
| 26 | C-Dogs | 0 | 0 | 0 | 0 | 0 | 0 |

| Dog Number | Group | Observer 3 (Resident) | | | | | |
| --- | --- | --- | --- | --- | --- | --- | --- |
|  |  | Compliance Score Flexion 1 | Compliance Score Flexion 2 | Compliance Score Flexion 3 | Compliance Score Extension 1 | Compliance Score Extension 2 | Compliance Score Extension 3 |
| 1 | CCL-Dogs | 0 | 0 | 0 | 0 | 0 | 0 |
| 2 | CCL-Dogs | 1 | 0 | 0 | 1 | 0 | 0 |
| 3 | CCL-Dogs | 0 | 0 | 1 | 0 | 2 | 1 |
| 4 | CCL-Dogs | 4 | 4 | 4 | 4 | 4 | 4 |
| 5 | CCL-Dogs | 0 | 0 | 0 | 1 | 1 | 1 |
| 6 | CCL-Dogs | 0 | 0 | 0 | 2 | 1 | 1 |
| 7 | CCL-Dogs | 0 | 0 | 0 | 0 | 0 | 0 |
| 8 | CCL-Dogs | 0 | 0 | 0 | 0 | 0 | 0 |
| 9 | CCL-Dogs | 0 | 0 | 0 | 0 | 0 | 0 |
| 10 | CCL-Dogs | 0 | 0 | 0 | 0 | 0 | 0 |
| 11 | CCL-Dogs | 0 | 0 | 0 | 1 | 1 | 1 |
| 12 | CCL-Dogs | 0 | 0 | 0 | 0 | 0 | 0 |
| 13 | CCL-Dogs | 0 | 0 | 0 | 0 | 0 | 0 |
| 14 | CCL-Dogs | 0 | 0 | 0 | 1 | 1 | 1 |
| 15 | CCL-Dogs | 0 | 0 | 0 | 0 | 0 | 0 |
| 16 | C-Dogs | 0 | 0 | 0 | 0 | 0 | 0 |
| 17 | C-Dogs | 0 | 0 | 0 | 0 | 0 | 0 |
| 18 | C-Dogs | 0 | 0 | 0 | 0 | 0 | 0 |
| 19 | C-Dogs | 0 | 0 | 0 | 0 | 0 | 0 |
| 20 | C-Dogs | 0 | 0 | 0 | 0 | 0 | 0 |
| 21 | C-Dogs | 0 | 0 | 0 | 0 | 0 | 0 |
| 22 | C-Dogs | 0 | 0 | 0 | 0 | 0 | 0 |
| 23 | C-Dogs | 0 | 0 | 0 | 0 | 0 | 0 |
| 24 | C-Dogs | 0 | 0 | 0 | 0 | 0 | 0 |
| 25 | C-Dogs | 0 | 0 | 0 | 0 | 0 | 0 |
| 26 | C-Dogs | 0 | 0 | 0 | 0 | 0 | 0 |
